# Pediatric Masked Mastoiditis Associated with Multiple Intracranial Complications

**DOI:** 10.1155/2015/897239

**Published:** 2015-06-28

**Authors:** Charalampos Voudouris, Ioannis Psarommatis, Ioannis Nikas, Dimitrios Kafouris, Konstantina Chrysouli

**Affiliations:** ^1^ENT Department, “P. & A. Kyriakou” Children's Hospital of Athens, Athens, Greece; ^2^Imaging Department, “Agia Sophia” Children's Hospital of Athens, Athens, Greece

## Abstract

Masked mastoiditis is a distinct form of mastoiditis with little or no symptomatology, characterized by its potential to generate severe otogenic complications. Therefore, suspected masked mastoiditis should be diagnosed and treated without delay. This study reports a rare case of masked mastoiditis, manifested by multiple intracranial complications in an immunocompetent girl. The child exhibited headache and neurological symptomatology. Imaging studies revealed an epidural and a large cerebellar abscess and the patient was immediately treated with a triple antibiotic therapy. Mastoid surgery and drainage of the epidural abscess took place after the stabilization of the patient's neurologic status, on the 3rd hospitalization day. The cerebellar abscess was treated by craniectomy and ultrasound-guided needle aspiration in the 3rd week of hospitalization. The girl was finally discharged in excellent condition. Two years later, she is still in good health, without otological or neurological sequelae. Masked mastoiditis is an insidious disease which requires increased clinical awareness and adequate imaging. Should clinical and/or radiological findings be positive, mastoidectomy must follow in order to prevent severe otogenic complications that can be triggered by masked mastoiditis.

## 1. Introduction

Masked mastoiditis is a rare yet distinct clinical entity, reported by many authors in the spectrum of intratemporal complications of otitis media [1, 2]. It may be defined as a suppurative subclinical process of the mastoid cavity, involving both the mucosa and the bony structures of the mastoid air-cell system. An incompletely healed acute otitis media and the obstruction of the aditus-ad-antrum by mucosal edema and granulation tissue are believed to represent the underlying pathogenetic mechanism. Under these circumstances, masked mastoiditis can potentially result in an effusion-free middle ear cavity, as the latter drains through the eustachian tube, combined with an active chronic inflammation within the blocked mastoid air-cell system [3]. The mastoid inflammation may last weeks, months, or even years before healing occurs—with or without surgical intervention—or its complications become symptomatic [4].

We report on an unusual case of pediatric masked mastoiditis presented with central nervous system (CNS) symptomatology due to multiple intracranial suppurative complications. Hopefully, this report will provide a better insight to clinicians in order to better understand the clinical behaviour and the aggressiveness of this disease, so that new awareness leads to improved diagnostic and therapeutic approach.

## 2. Case Report

An 8-year-old girl with no past medical history was taken to the emergency department with disequilibrium and gait instability, confusion, dysarthria, and vomiting, all manifested within the last 24 hours. An episode of acute otitis media on her right ear two weeks before treated with an oral antibiotic (amoxicillin 90 mg/kg/24 h for 10 days), along with a one-week history of vague occipitoparietal headache, was mentioned by her parents.

During her physical examination, spontaneous nystagmus, pathologic smooth pursuit eye movement, adiadochokinesia, and positive Romberg test were observed. The child developed fever up to 39°C in the first days after the diagnosis of acute otitis media, but she had no fever in the last week. Otoscopy revealed a near normal tympanic membrane, showing only minor redness with a clear lighting reflex. Type A tympanograms were recorded on both ears. Neurologic and neurosurgical consultations followed and the girl was admitted to the pediatric department and referred to imaging.

**Figure 1:**
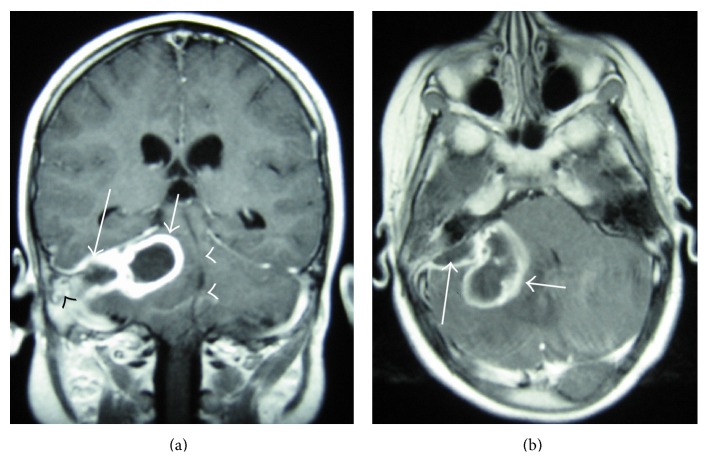
Coronal (a) and axial (b) MRI sections with contrast on admission, showing a large cerebellar abscess on the right hemisphere (short arrows) coexisting with an epidural abscess of the posterior fossa (long arrows) and inflammatory tissue within the mastoid cavity ((a), black arrowhead). The abscesses exert mass effect against the middle-line structures, causing their displacement to the left ((a), white arrowheads).

**Figure 2:**
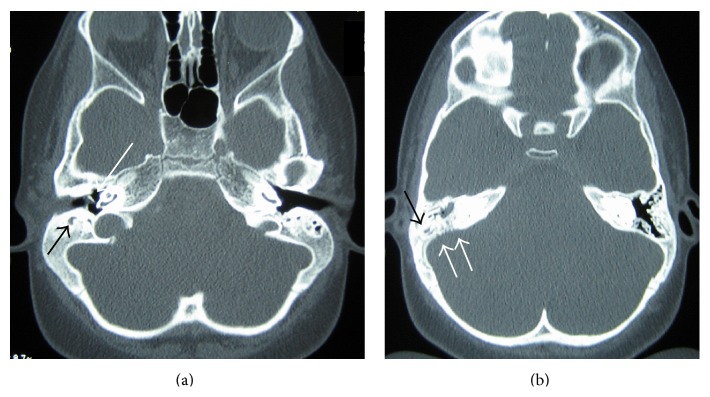
(a) and (b), axial sections from the preoperative temporal bone CT (without contrast) of the presented case. (a) Section through the mesotympanum showing the poorly developed mastoids on both sides. The right mastoid cells are filled with soft tissue (short arrow), while the middle ear cavity is free of effusion (long arrow). (b) Section at the level where the bone erosion can be observed (double arrows). Again, the right mastoid air cells and antrum are cloudy (short arrow).

Brain magnetic resonance imaging (MRI) revealed an impressive in size abscess cavity, localized at the right cerebellar hemisphere and extending towards the cerebellopontine angle. A second, epidural abscess, localized in the superior-posterior part of petrous bone was also observed (Figure 1). The right mastoid was poorly developed and opaque, indicating that it was occupied with inflammatory content. Magnetic resonance venography yielded no findings of thrombophlebitis. Finally, temporal bone computed tomography (CT) without contrast was performed to provide the anatomic relations of the abscesses with the bony structures. Apart from that, CT scans also disclosed the weakened point of the posterior tegmen of the right mastoid, from where the abscess had probably arisen (Figure 2).

The patient was immediately given triple intravenous antibiotic therapy (ceftriaxone, vancomycin, and clindamycin) and dexamethasone. Mastoid surgery and drainage of the epidural abscess took place after the stabilization of the patient's neurologic status, on the 3rd day of the girl's hospitalization. Myringotomy revealed an effusion free middle ear. Mastoid cells and antrum were full of edematous mucosa and inflammatory granulation tissue, causing a substantial block at the level of aditus. Most of this tissue was removed and an unobstructed communication between middle ear and antrum was established. While drilling at the posterosuperior part of the mastoid tegmen, the epidural abscess was cut open and the purulent exudate filled the surgical field. Specimens were taken for microbiological cultures. The communication between the two compartments (epidural and mastoid) was further opened to a size of approximately 1/2 cm^2^. Mastoidectomy was concluded by closing in layers and leaving a rubber drain within the mastoid cavity.

**Figure 3:**
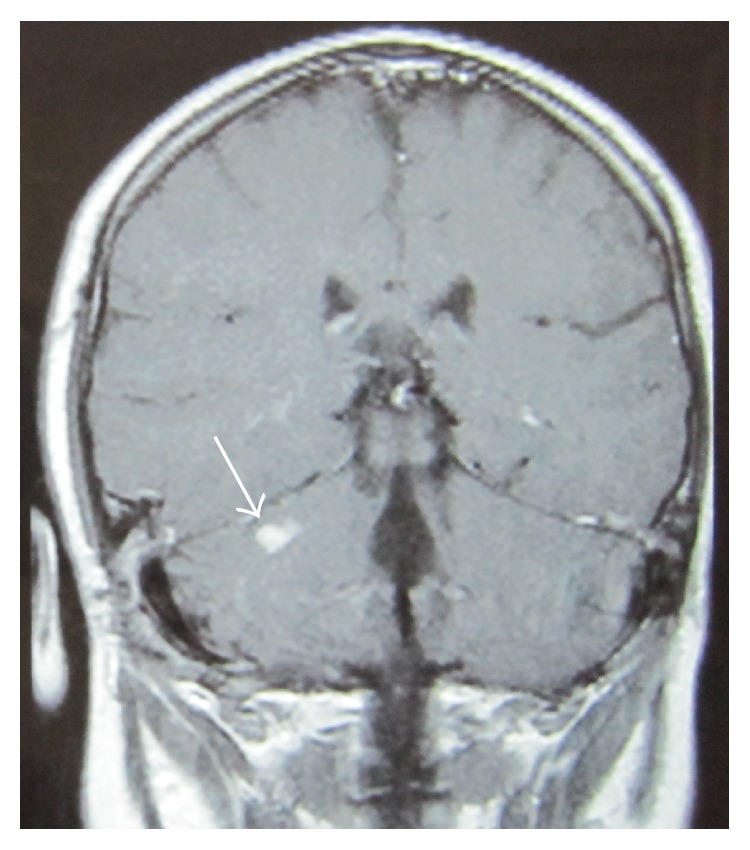
Only a small residual lesion (arrow) is observed, two months after the drainage of the cerebellar abscess.

Drainage of the cerebellar abscess through a craniectomy and ultrasound-guided needle aspiration and instillation of antibiotic solution were successfully performed by the neurosurgical team on the 3rd week of hospitalisation. Cultures from both abscesses were negative and the patient remained under the initial empiric antibiotic treatment. Repeat aspiration of the cerebellar abscess was not required. The girl recovered and was finally discharged from the hospital in excellent condition (Figure 3). During a two year follow-up, the patient showed no otological or neurological sequelae and she continues to be in good health.

## 3. Discussion

There are several reports of masked mastoiditis in the literature, most of which describe single intracranial or intratemporal complications [3, 5–8]. In contrast, only one case of masked mastoiditis manifested with multiple intracranial complications has been reported so far [3].

In the case described above were diagnosed two separate intracranial complications (epidural and brain abscesses) that endangered the life of the girl. Notably, these life-threatening complications happened in an immunocompetent child, who did not belong to any of the previously reported high risk populations for developing masked mastoiditis [3, 9].

Our patient was characterized by otoscopic, tympanometric, imaging, and surgical findings confirmatory of a clear middle ear (Figure 2). It becomes evident that masked mastoiditis and severe intracranial complications can occur even in the absence of disease in the middle ear cavity.

More than one factor may account for the development of masked mastoiditis, such as presence of middle ear pathology, middle ear anatomy, and the conditions of the microenvironment within the mastoid cavity, virulence of bacteria involved in suppurative otitis media, immunodeficiencies, use of systemic antibiotics, and so forth. Unfortunately, as yet we do not know the extent of their contributions to the development of masked mastoiditis. No matter what the cause is, it can be said with certainty that pediatric masked mastoiditis represents a distinct and potentially aggressive clinical entity, which can give rise to life-threatening complications.

Taking into consideration the insidious nature of the disease, increased clinical awareness coupled with low threshold for ordering the proper imaging studies is necessary for a prompt and safe diagnosis. Should clinical and/or radiological findings be positive, myringotomy and mastoidectomy must follow. The above recommendations are deemed mandatory in order to prevent the ominous otogenic complications that can be triggered by masked mastoiditis.

It can be argued that the patient's brain abscess could have been drained earlier, but the attending neurosurgical team deemed fit to initially manage the abscess conservatively under close supervision and repeated MRI studies. It also worth noting the short time interval that elapsed between the episode of acute otitis media and the formation of a mature brain abscess (just two weeks), although a detailed discussion about the pathogenesis of brain abscesses is beyond the scope of this paper.

## 4. Conclusions

- Pediatric masked mastoiditis is a distinct, aggressive, and potentially fatal clinical entity.
- Middle ear cavity does not necessarily participate in this specific form of mastoiditis.
- An intact tympanic membrane with near-normal appearance does not invariably guarantee a mastoid cavity free of disease.
- A high level of clinical awareness coupled with fast reflexes to the proper imaging studies is vital for early diagnosis.
- Any patient with a positive history of persistent middle ear symptoms and headache, with or without otorrhea, should have an ENT examination. If masked mastoiditis cannot otherwise be excluded, a CT scanning should be considered.
- If clinical and/or imaging findings are positive, middle ear exploration and mastoidectomy must follow.
